# Bidirectional pressure: a mini review of ventilator-lung-kidney interactions

**DOI:** 10.3389/fphys.2024.1428177

**Published:** 2024-06-20

**Authors:** Avnee Kumar, Katie Epler, Sean DeWolf, Laura Barnes, Mark Hepokoski

**Affiliations:** ^1^ VA San Diego Healthcare System, San Diego, CA, United States; ^2^ Division of Pulmonary and Critical Care and Sleep Medicine, University of California San Diego, San Diego, CA, United States

**Keywords:** AKI (acute kidney injury), lung injury, mechanical ventilatioin, lung-kidney crosstalk, acute respiratory distress syndrome

## Abstract

Acute kidney injury and respiratory failure that requires mechanical ventilation are both common complications of critical illnesses. Failure of either of these organ systems also increases the risk of failure to the other. As a result, there is a high incidence of patients with concomitant acute kidney injury and the need for mechanical ventilation, which has a devasting impact on intensive care unit outcomes, including mortality. Despite decades of research into the mechanisms of ventilator-lung-kidney interactions, several gaps in knowledge remain and current treatment strategies are primarily supportive. In this review, we outline our current understanding of the mechanisms of acute kidney injury due to mechanical ventilation including a discussion of; 1) The impact of mechanical ventilation on renal perfusion, 2) activation of neurohormonal pathways by positive pressure ventilation, and 3) the role of inflammatory mediators released during ventilator induced lung injury. We also provide a review of the mechanisms by which acute kidney injury increases the risk of respiratory failure. Next, we outline a summary of the current therapeutic approach to preventing lung and kidney injury in the critically ill, including fluid and vasopressor management, ventilator strategies, and treatment of acute kidney injury. Finally, we conclude with a discussion outlining opportunities for novel investigations that may provide a rationale for new treatment approaches.

## Introduction

Mechanical ventilation is an independent risk factor for the development of acute kidney injury (AKI) (van den Akker et al., 2013). Conversely, AKI independently doubles the risk of respiratory failure requiring mechanical ventilation (Metnitz et al., 2002; Waikar et al., 2007). As a result, roughly 75% of patients with AKI will be exposed to mechanical ventilation concomitantly during their intensive care unit (ICU) stay (Uchino et al., 2005). The consequences of combined AKI and the need for mechanical ventilation are severe, as mortality has been shown to increase 4-6-fold compared to AKI or respiratory failure alone (Chertow et al., 1995; Uchino et al., 2005; Vemuri et al., 2022). Moreover, AKI during mechanical ventilation is associated with prolonged hospitalizations, increased ICU stays, and increased ventilator days (Vemuri et al., 2022). Pre-clinical and clinical studies have described cellular, molecular, and mechanical crosstalk between the lung and kidney as mechanisms to explain the synergistic impact on mortality. Unfortunately, several knowledge gaps remain which have precluded the development of novel therapeutic approaches, and treatment remains primary supportive. Specifically, there is a paucity of data regarding the impact of mechanical ventilation on intrarenal physiology, as well as the relationship between alterations in kidney function and renal parenchymal damage. The exact mediators involved in inflammatory crosstalk between the lung and kidney that are modifiable is also unclear. In this review, we describe our current understanding of the mechanisms involved in ventilator-lung-kidney interactions. We also provide a critical review of our current treatment approaches to preventing lung and kidney injury during mechanical ventilation and AKI, respectively. Finally, we review current gaps in knowledge and opportunities for novel investigations that may lead to the development of new therapeutic strategies that are lifesaving.

## Mechanisms of AKI due to mechanical ventilation

**FIGURE 1:**
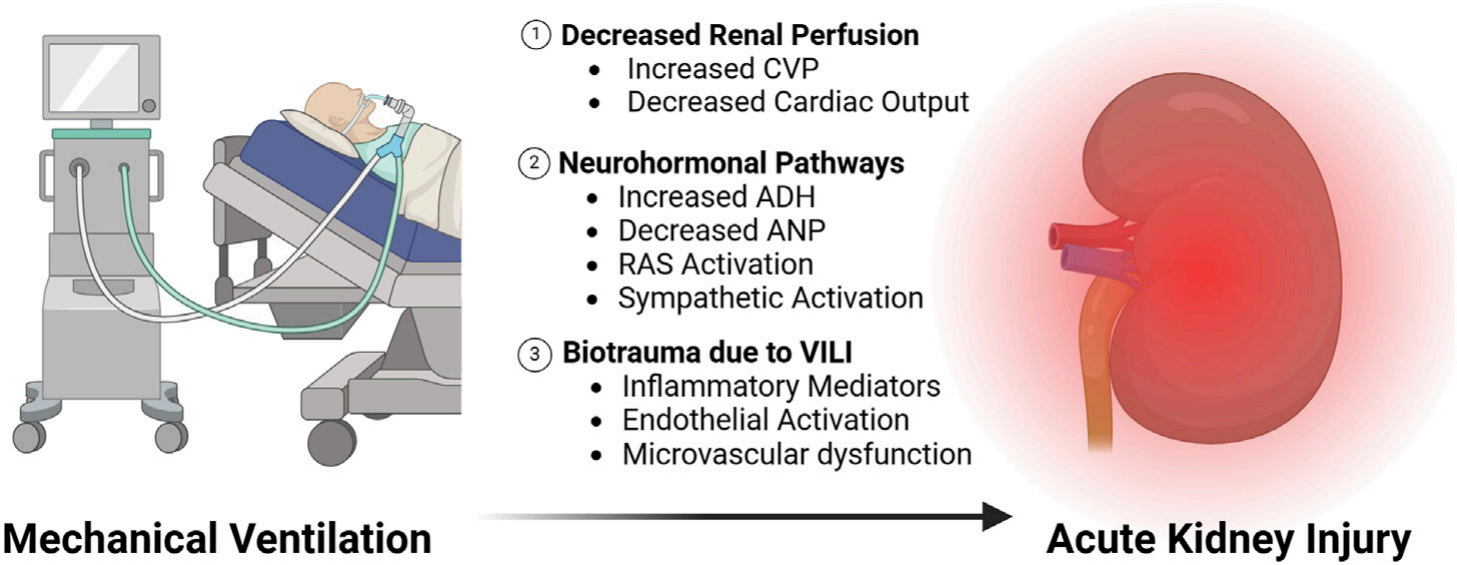
Mechanisms of AKI due to mechanical ventilation.

In 2013, Van den Akker et al. performed a systematic review and meta-analysis that included 31 studies focused on the relationship between the use of mechanical ventilation and subsequent AKI (van den Akker et al., 2013). Their analysis showed that invasive mechanical ventilation independently increases the odds of AKI 3-fold (van den Akker et al., 2013). We conducted a more recent analysis that found that the incidence of AKI during mechanical ventilation remains incredibly high at 39% (Vemuri et al., 2022). Interestingly, our study also found that most AKI cases occurred 1–3 days after mechanical ventilation was initiated, which is suggestive of a potential causal relationship. In this section, we review our current understanding of the mechanisms by which mechanical ventilation impacts kidney function and may lead to AKI (Figure 1).

The best described mechanism to explain the high incidence of AKI during mechanical ventilation involves the systemic hemodynamic effects of positive pressure ventilation. Over 75 years ago, Drury et al. demonstrated that increasing levels of continuous positive airway pressure (CPAP) correlated inversely with urea clearance in healthy volunteers (Drury et al., 1947). To explain these findings, the authors proposed that an increase in intrathoracic pressure during mechanical ventilation led to decreases in venous return and cardiac output, which led to a decrease in renal perfusion. This hypothesis was confirmed in subsequent studies in canines where increasing levels of CPAP or positive end expiratory pressure (PEEP) correlated with a decrease in renal perfusion (Hall et al., 1974; Qvist et al., 1975). Furthermore, improving cardiac output with fluid administration was shown to restore renal blood flow (Qvist et al., 1975). Beyond the effects on cardiac output, positive pressure also impacts renal perfusion through increased central venous pressure (CVP) and venous congestion. Renal perfusion pressure is equal to mean arterial pressure (MAP)–CVP. Therefore, as CVP is increased with positive pressure ventilation, renal perfusion will be decreased for a given MAP (Sun et al., 2022). Increasing CVP and PEEP both correlate with decreased glomerular filtration rate (GFR) in human subjects (Farge et al., 1995; Sun et al., 2022).

Neurohormonal pathways activated by positive pressure breathing have also been implicated in the alterations in kidney function that occur during mechanical ventilation. Fewell and Bond were among the first to implicate these mechanisms by showing that renal denervation prior to positive pressure ventilation improved urine output and GFR (Fewell and Bond, 1979). In subsequent studies, activation of the renin angiotensin system (RAS) (Kaczmarczyk et al., 1992), increased release of antidiuretic hormone (ADH) (Bark et al., 1980; Farge et al., 1995), also known as vasopressin, and decreases in atrial natriuretic peptide (ANP) (Ramamoorthy et al., 1992) were found to occur during mechanical ventilation in both human and animal models. These pathways are believed to further decrease renal blood flow and GFR through prerenal vasoconstriction, in addition to promoting sodium retention. The exact mechanisms that contribute to activation of these neurohormonal pathways have not been fully elucidated, though the impact of intrathoracic pressure on atrial stretch, carotid baroreceptors, and sympathetic nervous system outflow are all likely to contribute (Fewell and Bond, 1979).

Finally, inflammatory crosstalk between the injured lung and kidney during injurious mechanical ventilation with high tidal volumes and high airway pressures has been found to cause renal tubular injury and cell death (Choi et al., 2003; Imai et al., 2003; Hepokoski et al., 2017). In a landmark study, Imai et al. demonstrated that serum from rabbits who developed ventilator induced lung injury (VILI) due to high tidal volume ventilation caused renal tubule cell apoptosis *in vitro* and in healthy rabbits *in vivo* (Imai et al., 2003). These findings provided a direct link between systemic inflammatory mediators generated by lung injury and downstream renal consequences (i.e., biotrauma). Subsequent studies from our group and others found that VILI leads to endothelial inflammation (Hepokoski et al., 2017) and microvascular dysfunction in the kidney (Choi et al., 2003), and the impact of VILI on the kidney remains an active area of investigation.

## Mechanisms of lung injury due to AKI

**FIGURE 2:**
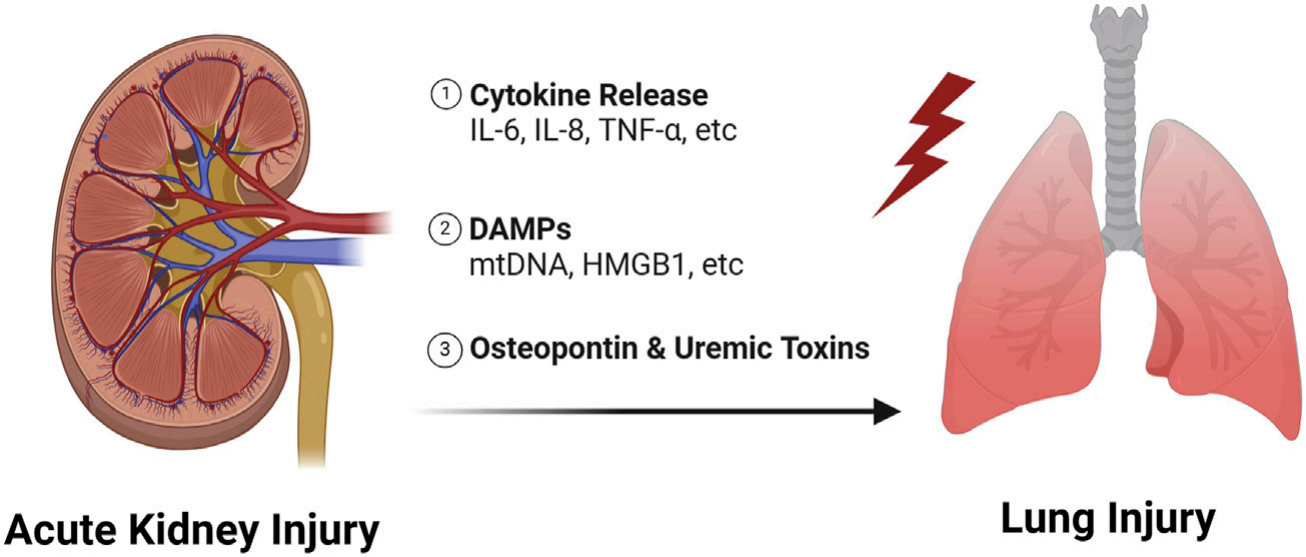
Mechanisms of lung injury due to AKI.

Respiratory failure is one of the leading causes of death due to AKI, particularly when mechanical ventilation is required (Chertow et al., 1995). We showed recently that AKI during mechanical ventilation was associated with impaired gas exchange measured by the ratio of the partial pressure of oxygen in arterial blood to the fraction of inspiratory oxygen concentration (Vemuri et al., 2022). Interestingly, we also found that AKI was associated with a deleterious impact on lung mechanics, as patients with AKI had decreased lung compliance, increased driving pressures, and increased plateau pressures compared to mechanically ventilated patients without AKI. Volume overload in the setting of oliguria would certainly contribute to these findings, but we showed that the effects of AKI on both lung mechanics and gas exchange remained significant in patients who had a net negative cumulative fluid balance. These results suggest that other mechanisms, such as inflammatory crosstalk from the kidney to lung, may be playing a role. In this section, we review mediators of lung injury due to AKI that have the potential to be modifiable (Figure 2).

Lung injury as a consequence of AKI has been described as early as the 1950s (Bass and Singer, 1950), and was originally thought to be due to decreased renal function resulting in the accumulation of uremic toxins. Recently, Yabuuchi et al. showed that indoxyl sulfate, a uremic toxin, accumulates in the lung after AKI and leads to decreased aquaporin five expression (Yabuuchi et al., 2016). Aquaporin five is used by alveolar epithelial cells to clear water from the alveolar space, thus downregulation of aquaporin may be an important mechanism in AKI induced pulmonary edema, especially in pulmonary edema that is resistant to diuretics. Interestingly, Hassoun et al. compared the effects of an ischemia-reperfusion (IR)-AKI model to bilateral nephrectomy and found that IR caused more severe lung injury (Hassoun et al., 2007). Therefore, lung injury due to AKI is less likely to be due to loss of kidney function alone and inflammatory mediators released from the injured kidney likely contribute.

Over the past few decades, there has been a growing literature which has revealed a complex set of inflammatory mediators that are generated during AKI and contribute to lung injury (Faubel and Edelstein, 2016). The pathophysiology is believed to involve the release of cytokines, damage associated molecular patterns (DAMPs), and other pro-inflammatory ligands into the circulation which then travel to the lung and bind to receptors on lung tissue (Faubel and Edelstein, 2016; Klein et al., 2018; Lee et al., 2018; Alge et al., 2021; Herrlich, 2022). These ligand-receptor interactions initiate pro-inflammatory responses that lead to increased vascular permeability, immune cell infiltration and non-cardiogenic pulmonary edema. Neutrophilic inflammation is a hallmark of most forms of lung injury (Ishii et al., 2010; Masso-Silva et al., 2022), but recruitment of T-cells to the lung during AKI is also particularly important. T-cells have been found to accumulate in the lung 24-h after IR-AKI, and pulmonary edema is mitigated in T-cell deficient mice (Lie et al., 2012).

Interleuken-6 (IL-6) is perhaps the best described mediator of lung injury due to AKI with data supporting the role of IL-6 mediated inflammation in both pre-clinical and clinical studies. For example, Klein et al. showed IL-6 knockout mice and wildtype mice treated with IL-6 blocking antibodies were protected from lung injury after IR-AKI, despite equivalent levels of renal dysfunction (Klein et al., 2008). Increased IL-6 concentrations are also associated with increased mortality and duration of mechanical ventilation in patients with acute respiratory distress syndrome (ARDS) and AKI (Simmons et al., 2004; Liu et al., 2009). Finally, other cytokines, including Interleukin-8 (IL-8) and tumor necrosis factor (TNF) have been shown to be increased in the circulation following AKI and contribute directly to lung inflammation (Kelly, 2003; Hoke et al., 2007; Liu et al., 2009; Ahuja et al., 2012; White et al., 2012).

DAMPs are endogenous molecules released from injured/dying cells during times of stress (Rosin and Okusa, 2011; Tolle and Standiford, 2013; Vénéreau et al., 2015; Meissner et al., 2019; DeWolf et al., 2022) and are believed to travel from the kidney to the lung where they exert pro-inflammatory effects (Faubel and Edelstein, 2016; Hepokoski et al., 2018; Hepokoski et al., 2021; Alge et al., 2021; Hepokoski and Singh, 2022; Herrlich, 2022; Komaru et al., 2024). Recently, we showed that mitochondrial DNA is one such DAMP that is increased in the circulation of mice after IR-AKI (Hepokoski et al., 2021), and mitochondrial DNA is associated with lung injury in human subjects with COVID-19 (Hepokoski et al., 2022). We also showed that kidney mitochondrial DAMPs contribute directly to metabolic changes consistent with mitochondrial dysfunction in the lung (Hepokoski et al., 2021). DeWolf et al. showed recently that necrotic slurry generated from injured human primary renal tubular epithelial cells causes increased inflammation in human microvascular endothelial cells *ex-vivo* by increasing cytokine/adhesion molecule production, mitogen-activated protein kinases (MAPKs) and nuclear factor-κβ (NF- κβ) activation, and vascular permeability (DeWolf et al., 2024). High mobility group box 1 (HMGB1), another well-established DAMP, has also been shown to play key roles in AKI-induced lung injury in rodent models via activation of toll-like receptor 4 (Doi et al., 2014; Zhao et al., 2015).

Finally, Khamesi et al. demonstrated that osteopontin, an immune regulatory molecule (Rittling and Singh, 2015; Icer and Gezmen-Karadag, 2018; Jia et al., 2024), is produced in the kidney after AKI and travels to the lung to worsen lung injury (Khamissi et al., 2022). We highlight this elegant paper because it is the first to provide definitive evidence that the kidney is the source of pro-inflammatory molecules that exert effects in the lung after AKI. The cellular source of other cytokines and DAMPs, including those mentioned above, remains to be elucidated.

## Current approach to managing ventilator-lung-kidney interactions

The initial approach to preventing AKI due to mechanical ventilation involves counteracting the systemic hemodynamic effects of positive pressure via a combination of fluids and vasopressors. However, the amount of fluids vs. vasopressors and the optimal vasopressors for kidney protection have not been clearly established. Furthermore, the Fluids and Catheters Treatment Trial (FACTT) in 2006 showed that patients treated with a conservative fluid management strategy spent fewer days requiring mechanical ventilation, in addition to a non-significant trend towards lower dialysis requirements (Wiedemann et al., 2006). In terms of the type of fluid management, evidence suggests that balanced crystalloids decrease the need for renal replacement therapy or persistent renal dysfunction (Semler et al., 2018). It should be noted that only 1/3rd of patients included in this study required mechanical ventilation, and the use of balanced crystalloids as compared to normal saline did not impact ventilator free days. Still, fluid resuscitation with balanced crystalloids is used for those patients that are volume depleted. In general, a conservative fluid management strategy is followed after adequate resuscitation is achieved despite a lack of data to support an improvement in hard kidney outcomes.

Norepinephrine is considered the first line vasopressor in adult critically ill patients, but recent studies have suggested that combination vasopressors, such as β-agonists with angiotensin II (Tumlin et al., 2018), may improve renal outcomes. The impact of specific vasopressors alone and in combination remains an active area of investigation. As noted previously, increasing CVP due to mechanical ventilation has a deleterious impact on renal perfusion and increasing CVP is associated with AKI (Sun et al., 2022). It is tempting to suggest that patients on mechanical ventilation should have a higher MAP goal to counteract the effects of high CVP. However, there is a paucity of data to support this notion currently and maintaining a MAP greater than 65 mmHg is generally believed to provide adequate renal perfusion.

Choosing optimal ventilator settings for lung and kidney protection is another important consideration in preventing both ventilator induced lung and kidney injury. The current approach to ventilator management is based on the 2000 Acute Respiratory Distress Syndrome Network (ARDSNet) trial which showed that patients with ARDS treated with open lung protective ventilation (low tidal volumes and high PEEP) had an increase in survival and renal failure free days (Acute Respiratory Distress Syndrome et al., 2000). Interestingly, the magnitude of improvement in renal outcomes with lung protective ventilation outweighed those in the cardiovascular, hepatic, neurologic, and hematologic systems (Ranieri et al., 2000). While low tidal volume ventilation is clearly renal protective, there is some controversy regarding the optimal PEEP. As reviewed above, pre-clinical and clinical studies have suggested that high PEEP induces renal dysfunction, yet other studies have shown no correlation between risk of AKI and PEEP (van den Akker et al., 2013; Almonacid-Cardenas et al., 2023; Basse et al., 2023). More recently, studies have shown that the driving pressure (plateau pressure-PEEP), which accounts for lung compliance, is a better predictor of patient outcomes than tidal volume or PEEP alone (Amato et al., 2015). However, the relationship between driving pressure and AKI has not been clearly established, and the optimal PEEP vs. driving pressure remains to be established. For now, low tidal volume ventilation with 6–8 cc/kg based on ideal body weight is universally employed for most patients, but optimal lung and kidney protective ventilator settings are likely not “one size fits all”. Studies focused on determining precision, patient-specific ventilator settings are ongoing (Beitler et al., 2022).

Once AKI has occurred treatment is focused on mitigating further injury, avoiding nephrotoxic medications, and appropriate dosing of active medications. In a 2016 trial in ICU patients that included 80% of patients who were on mechanical ventilation, early renal replacement therapy (RRT) did not improve mortality. It should also be noted that about half of the patients in the delayed group never required RRT (Gaudry et al., 2016). It is possible that early RRT may improve outcomes in patients who will ultimately need RRT, but the identification of those patients is challenging. Finally, the use of extracorporeal carbon dioxide removal (ECCO_2_R) and extracorporeal membrane oxygenation (ECMO) are increasingly used to support gas exchange in the critically ill, but there is limited data regarding the role of these advanced therapies in lung and kidney protection.

## Discussion

The studies outlined above have predominately focused on the impact of mechanical ventilation on renal perfusion and GFR, but how these functional changes translate to “structural AKI” with parenchymal damage remains unclear. This concept is important, as a decrease in renal perfusion or oxygen delivery to the kidney does not necessarily lead to tubule injury and AKI if oxygen utilization also decreases. Tubule transport work, particularly sodium reabsorption in the proximal tubule, utilizes more oxygen and ATP than any other kidney function (Wang et al., 2010; Bhargava and Schnellmann, 2017), and tubule transport work is directly related to GFR. Thus, in the healthy state, oxygen consumption decreases in the setting of low renal perfusion, which protects the kidney from hypoxia and tubule damage (Hansell et al., 2013). Therefore, it is possible that the decreased GFR observed during mechanical ventilation could be protective. It is also possible that attempts to restore GFR via fluid administration or vasopressors could be harmful if the result is an increase in tubule transport work. Lack of understanding of the relationship between intrarenal physiological changes during mechanical ventilation and AKI remains a major knowledge gap that has precluded the development of novel treatment approaches.

As outlined above, mechanical ventilation is associated with both decreased renal blood flow and increased sodium reabsorption. These changes would be predicted to both decrease oxygen delivery and increase oxygen consumption due to increased tubule transport work, which may lead to hypoxia, metabolic stress, and cellular damage. Intriguingly, Glucagon-like peptide-1 (GLP-1) agonists have been shown to impact renal physiology in a manner that may counteract the deleterious effects of mechanical ventilation on the kidney. For example, the GLP-1 agonist, exenatide, has been found to increase renal blood flow via intrarenal vasodilation, in addition to inhibiting sodium reabsorption at the level of the proximal tubule, which could offer renal protection (Thomson et al., 2013; Thomson et al., 2017). Supporting this notion, GLP-1 agonists have been shown to decrease renal oxidative stress and histological kidney injury in a pre-clinical model of cisplatin-induced AKI (Katagiri et al., 2013). Other treatments targeting kidney metabolism, such as nicotinamide adenine dinucleotide (NAD+) augmentation, have also been found to prevent AKI in animal models and phase-1 clinical trials (Poyan et al., 2018). These treatments may be a better alternative than the current approach of preserving renal blood flow via fluid administration or vasopressors, and investigations in this area are warranted.

Finally, prevention of lung injury due to AKI and *vice versa* has been limited by challenges in early detection. As mentioned previously, inhibitors of inflammatory mediators, such as IL-6 (Klein et al., 2008), have shown promising results in preclinical studies. The IL-6 inhibitor, tocilizumab, has also shown some benefit in the treatment of COVID-19 (Group et al., 2021). Unfortunately, most inflammatory mediators promote lung and kidney injury at time points prior to clinical detection of AKI. Importantly, novel biomarkers of kidney injury, such as kidney injury molecule-1 (KIM-1) and insulin like growth factor binding protein 7 (IGFBP7) have been shown to predict moderate-severe AKI several hours prior to increased serum creatinine, and serial measurements of IGFBP7 have been shown to respond to clinical management (Fiorentino et al., 2020). These and other novel biomarkers may allow for earlier administration of anti-inflammatory treatments in patients who would benefit. They also could be used as tools to alert clinicians to patients who are at increased risk of AKI and VILI, which would allow them to respond with ventilator changes or the early application of advanced therapies, such as ECCO_2_R or ECMO.

## Conclusion

In conclusion, AKI during mechanical ventilation, and *vice versa*, are common complications of critical illness that are associated with unacceptably high morbidity and mortality. Unfortunately, there is minimal evidence currently available to support treatments beyond lung protective ventilation with low tidal volumes. To develop novel therapeutic approaches, we must advance our understanding of the relationship between alterations in kidney physiology during mechanical ventilation and structural AKI. The mechanisms by which AKI impacts lung mechanics and gas exchange beyond volume overload also warrants immediate investigation. Finally, translational studies utilizing novel biomarkers to predict reciprocal need for mechanical ventilation and AKI may lead to earlier recognition, clinical characterization, and treatment. Only with novel, mechanistic research are new treatments likely to be discovered.
